# Risk of Spontaneous Preterm Birth in a Subsequent Pregnancy After Full Dilatation Caesarean Birth: A Nationwide Cohort Study

**DOI:** 10.1111/1471-0528.18225

**Published:** 2025-05-28

**Authors:** Sara Acker, Catharina Hein Hoffmann, Lea Kirstine Hansen, Julie Glavind, Maria Jeppegaard, Lone Krebs

**Affiliations:** ^1^ Department of Gynaecology and Obstetrics Copenhagen University Hospital – Amager and Hvidovre Hvidovre Denmark; ^2^ Department of Obstetrics and Gynaecology Aarhus University Hospital Aarhus Denmark; ^3^ Department of Clinical Medicine Aarhus University Hospital Aarhus Denmark; ^4^ Department of Gynaecology and Obstetrics Copenhagen University Hospital – Holbaek Denmark; ^5^ Department of Clinical Medicine University of Copenhagen Copenhagen Denmark

**Keywords:** caesarean, full dilatation, preterm birth, second‐stage caesarean

## Abstract

**Objective:**

To investigate the association between caesarean birth in the second stage of labour and the risk of spontaneous preterm birth in a subsequent pregnancy.

**Design:**

Nationwide register‐based cohort study.

**Setting:**

The Danish Medical Birth Registry and The Danish National Patient Register from 1997 to 2021.

**Population:**

Women with at least two consecutive births (index birth and subsequent birth) where the index birth was a term singleton.

**Methods:**

Women with spontaneous preterm subsequent birth were compared according to the mode of index birth, including vaginal, prelabour caesarean, first stage caesarean and second‐stage caesarean.

Statistical analysis was performed using multiple logistic regression.

**Main Outcome Measure:**

Spontaneous birth before 37 + 0 gestational weeks.

**Results:**

A total of 376 414 women met the inclusion criteria. Distribution of mode of index birth was vaginal 318 117 (84.5%), prelabour caesarean 15 373 (4.1%), first stage caesarean 37 547 (10.0%) and second‐stage caesarean 5377 (1.4%), respectively. The rate of subsequent preterm birth was 1.7%. Compared to vaginal birth, a second‐stage caesarean was associated with an increased risk of preterm birth in a subsequent pregnancy (adjusted odds ratio [aOR] 1.46, 95% confidence interval [CI] 1.21–1.77). Similarly, compared to first stage caesarean, a second‐stage caesarean was associated with an increased risk of preterm birth in the subsequent pregnancy, with an aOR of 1.41 (95% CI 1.15–1.74).

**Conclusion:**

Second‐stage caesarean in a previous term pregnancy is associated with an increased risk of spontaneous preterm birth in a subsequent pregnancy.

## Abstract 2

**Linked article:** This article is commented on by Ludlow pp. 1594–1595 in this issue. To view this article visit https://doi.org/10.1111/1471‐0528.18287.

**Abbreviations**
aOR: Adjusted Odds Ratio
BMI: Body Mass Index
CB: Caesarean Birth
CI: Confidence Interval
DMBR: Danish Medical Birth Register
GA: Gestational Age
OR: Odds Ratio
PPROM: Preterm Prelabour Rupture of Membranes
PTB: Preterm Birth
RR: Relative Risk
SD: Standard Deviation
sPTB: Spontaneous Preterm Birth

## 1 Introduction

Preterm birth (PTB) is the leading cause of neonatal mortality and lifelong disabilities in the surviving children [1]. Globally, the PTB rate is around 10% affecting approximately 14 million infants annually [2, 3]. Over the past decades, this rate has remained largely unchanged and has even increased in some regions [3]. In Denmark, the preterm birth rate is approximately 5%–6% [4]. Although significant progress has been made in understanding risk factors and mechanisms related to preterm labour, the cause of PTB remains unknown in more than 50% of cases [5, 6]. Even though a term delivery is considered a protective factor for spontaneous preterm birth (sPTB), recent research now indicates that a term caesarean birth (CB) in the second stage of labour may serve as an independent risk factor for sPTB in a subsequent pregnancy [7, 8, 9, 10, 11, 12, 13, 14, 15]. One hypothesis is that CB at full dilatation may lead to cervical trauma, leaving the cervix weaker in a subsequent pregnancy [11, 16, 17, 18].

The assumed association between CB and sPTB is crucial since rates of CB, including those at full dilatation, are increasing worldwide [19]. Most studies considering second‐stage CB as a risk factor for sPTB are based on populations outside the Nordic countries, where rates of CB and sPTB are substantially higher than in the Nordic countries [13, 20, 21]. A population‐based study from Canada, including 89 021 births, found second‐stage CB to be associated with sPTB < 32 weeks, with a relative risk (RR) of 2.1 [8]. These results are supported by findings from a recent Scottish population‐based study, which reported an odds ratio (OR) of 5.4 when comparing second‐stage CB to vaginal index birth [7]. Similarly, an Israelian study showed an adjusted OR (aOR) of 2.6, reflecting consistent findings across studies supporting this association [14]. Contrarily, studies from the Netherlands and Norway did not confirm the association, possibly because they were unable to distinguish between CB in the first and second stage of labour [22, 23]. The population‐based registries in Denmark present a unique opportunity to explore the relationship between mode of delivery and subsequent sPTB in a population with an inherently low baseline risk of sPTB. Furthermore, data from the Danish registers enables us to distinguish between birth modes in previous pregnancies and to subcategorise gestational age in the subsequent birth.

## 2 Objective

The aim of this study was to investigate in a large population‐based cohort the association between CB in the second stage of labour and the risk of sPTB in a subsequent pregnancy compared to vaginal birth and first stage CB.

## 3 Materials and Methods

The study was a register‐based cohort study based on data from the Danish Medical Birth Registry (DMBR) and the Danish National Patient Register (DNPR). We included nulliparous women delivering a singleton infant at term and at least one consecutive pregnancy registered in the DMBR during the study period from 01.01.1997 to 31.12.2021. The DMBR is a population‐based registry, which contains information on births after gestational age (GA) 22 + 0 weeks or below if the infant is recorded as liveborn. It was established in 1968, computerised since 1973, and provides validated data for quality improvement and research [24, 25]. The registry links together the personal ID number of mother, father and child, and includes information on maternal characteristics including age, weight, height and smoking status, as well as pregnancy‐related characteristics such as GA, birthweight and pregnancy outcome. The DNPR was established in 1977 and is the most extensive Danish health register. Information in the DNPR is based on codes from the International Statistical Classification of Diseases and Related Health Problems. Information regarding any procedure is based on the codes according to the Nordic Medico‐statistical Committee classification of surgical procedures.

**FIGURE 1:**
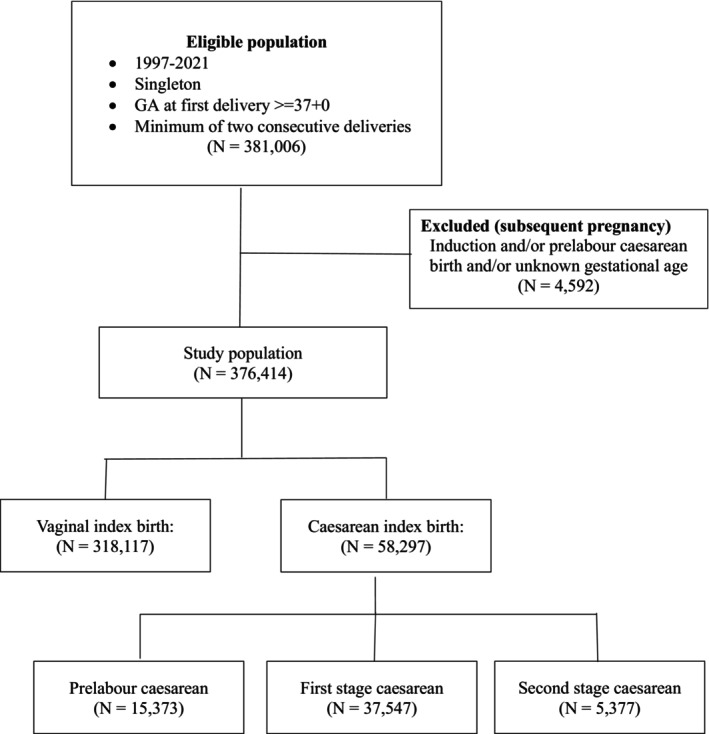
Flowchart of cohort inclusion and exclusion.

The first birth for women in the cohort was referred to as the ‘index birth’. The second consecutive birth was referred to as ‘subsequent birth’. All data regarding maternal characteristics, pregnancy‐ and birth outcomes were retrieved from the DMBR and the DNPR. We excluded women with an index birth with stillbirth, birth with GA < 37 + 0 weeks or GA unknown, application of cervical cerclage and unknown mode of birth. In data on subsequent births, we excluded women with induction of labour GA < 37 + 0 weeks, prelabour CB < 37 + 0 or unknown GA at birth, or birth before GA 22 + 0 weeks (Figure 1).

Based on data from the DMBR, we subdivided the study population into four exposure groups according to the mode of the index birth: vaginal birth, prelabour CB, first stage CB (< 10 cm dilatation) and second‐stage CB (full dilatation of the cervix). Women with no CB procedure code at their index birth were categorised as having a vaginal birth. Prelabour CB was identified from the DMBR according to intervention codes including elective CB and CB unscheduled prior to labour (Figure S1). CB without an attempt at operative vaginal birth or without a diagnosis of prolonged second stage of labour were categorised as first stage CB (< 10 cm dilatation) (Figure S1). Second‐stage CB was defined as a caesarean section performed after a failed vacuum or forceps delivery or a diagnosis of a prolonged second stage of labour (Figure S1).

Spontaneous preterm birth (sPTB) was defined as birth before GA 37 + 0 weeks with no information on induction of labour or CB before onset of labour. Outcomes were analysed in groups of gestation age (Table 2) and further analysed in subgroups according to GA at the time of the subsequent birth into four categories of preterm birth based on categories from the WHO [3]: extremely preterm (GA 22 + 0–27 + 6 weeks), very preterm (GA 28 + 0–31 + 6 weeks), late preterm (GA 32 + 0–36 + 6 weeks) or term birth (GA ≥ 37 + 0 weeks).

Covariates were selected based on known relation to sPTB and included maternal age, maternal body mass index (BMI), smoking status, hypertensive disorders, diabetic disorders and interpregnancy interval. The binary covariates were all recorded for the subsequent pregnancy. For adjustment, maternal age was dichotomised into maternal age ≤ 40 years or > 40 years, BMI was dichotomised into ≤ 30 kg/m^2^ or > 30 kg/m^2^.

Smoking status was categorised into non‐smoker (no use of tobacco during pregnancy), current smoker (either active smoker during pregnancy or stopped during pregnancy) or unknown. For the adjusted analysis, smoking status was dichotomised into smoker ‘yes’ or ‘no’.

Hypertensive disorders included essential hypertension, gestational hypertension and preeclampsia (ICD‐10 codes: DI109, DO11, DO13, DO14, DO139, DO140, DO141).

Diabetic disorders included both type 1 and type 2 diabetes, as well as gestational diabetes (ICD‐10 codes: DE10, DE11, D13, D14, DO24) [26]. For adjustment, both hypertensive disorders and diabetic disorders were dichotomised into ‘yes’ or ‘no’.

Interpregnancy interval was defined as the time from index to subsequent birth and was dichotomised into ≤ 12 months or > 12 months. Birthweight was dichotomised into ≤ 4000 g or > 4000 g.

Small‐for‐gestational age (SGA) was defined as birthweight ≤ −1.28 SD under mean according to gender and gestational age [27].

To reduce the impact of outliers, we applied cut‐offs for birthweight, maternal height, weight and BMI and categorised outliers as missing data. The following cut‐offs were used: birthweight < 100 g and > 6000 g, maternal height < 120 cm and > 220 cm, maternal weight < 40 kg and > 200 kg and maternal BMI < 10 kg/m^2^ and > 70 kg/m^2^.

In a secondary analysis, we evaluated the association between mode of index birth and complications during the subsequent pregnancy such as cerclage, preterm prelabour rupture of membranes (PPROM), placenta praevia and stillbirths.

### 3.1 Statistical Analysis

Data were analysed using RStudio version 4.4.1.

Demographic and clinical characteristics were reported per exposure group using counts (percentages) for categorical variables and mean (standard deviation) for numerical variables. Logistic regression was used to calculate crude and aOR and 95% confidence intervals (95% CI) using vaginal index birth or first stage CB as references. We adjusted for the following variables (recorded in the subsequent pregnancy): maternal age > 40 years, maternal BMI > 30 kg/m^2^, smoking status, hypertensive disorders, diabetic disorders and interpregnancy interval < 12 months.

The study was approved by the Danish Data Protection agency (PFI, Region Zealand); REG‐039‐2019.

## 4 Results

The study population included 376 414 women with two consecutive births during the study period (Figure 1). The overall CB rate was 15.3%. Characteristics of the population according to mode of birth in the index pregnancy are presented in Table 1. The groups did not differ regarding maternal height, weight, smoking status or interpregnancy interval. We found that women > 35 years were more likely to have a CB, with a slight overrepresentation of prelabour CB (6.7%), where women aged < 18 years were more likely to have a vaginal birth (Table 1).

**TABLE 1:** Demographic characteristics of the study population by mode of index birth.

	All, *n* = 376 414	Vaginal, *n* = 318 117	Prelabour CB, *n* = 15 373	First stage CB, *n* = 37 547	Second‐stage CB, *n* = 5377
Maternal age (years), mean (±SD)	27.5 (±4.2)	27.3 (±4.2)	28.6 (±4.5)	28.4 (±4.2)	28.2 (±4.1)
Maternal age categories, *n* (%)					
< 18	1945 (0.5)	1797 (0.6)	46 (0.3)	88 (0.2)	14 (0.3)
18–35	362 305 (96.3)	307 323 (96.6)	14 302 (93.0)	35 551 (94.7)	5129 (95.4)
> 35	12 164 (3.2)	8997 (2.8)	1025 (6.7)	1908 (5.1)	234 (4.4)
Maternal height (cm)^a^, mean (±SD), Missing *n* = 129 814	168 (±6)	168 (±6)	167 (±7)	166 (±7)	167 (±7)
Maternal weight (kg)^a^, mean (±SD) Missing, *n* = 129 719	68 (±15)	67 (±14)	69 (±17)	70 (±16)	67 (±14)
BMI (kg/m^2^)^a^, mean (± SD) Missing, *n* = 131 041	23.8 (±4.6)	23.6 (±4.5)	24.6 (±5.4)	25.1 (±5.2)	24.1 (±4.6)
BMI (kg/m^2^)^a^ categories Missing, *n* = 131 041					
< 25	174 086 (46.2)	149 052 (46.9)	7020 (45.7)	15 361 (40.9)	2653 (49.3)
25–29.9	47 358 (12.6)	38 107 (12.0)	2262 (14.7)	6176 (16.4)	813 (15.1)
≥ 30	23 929 (6.4)	18 091 (5.7)	1491 (9.7)	3959 (10.5)	388 (7.2)
Smoking, *n* (%)					
Non‐smoker	321 235 (85.4)	271 688 (85.4)	12 972 (84.4)	31 854 (84.8)	4721 (87.8)
Smoker	47 802 (12.7)	40 497 (12.7)	1932 (12.6)	4819 (12.8)	554 (10.3)
Unknown	7377 (2.0)	5932 (1.9)	469 (3.1)	874 (2.3)	102 (1.9)
GDM, *n* (%)	7446 (2.0)	5533 (1.7)	631 (4.1)	1149 (3.1)	133 (2.5)
DM1 + DM2, *n* (%)	1864 (0.5)	1202 (0.4)	237 (1.5)	377 (1.0)	48 (0.9)
Hypertensive disorders, *n* (%)	18 910 (5.0)	13 834 (4.3)	1533 (10.0)	3052 (8.1)	312 (5.8)
Gestational age (days), mean (±SD)	281 (±9)	281 (±9)	275 (±10)	284 (±9)	284 (±8)
Birthweight (g), mean (±SD) Missing, *n* = 2469	3510 (±470)	3495 (±449)	3393 (±585)	3664 (±549)	3752 (±463)
Weight groups (g), *n* (%) Missing, *n* = 2469					
≤ 4000 g	321 824 (85.5)	277 156 (87.1)	13 202 (85.9)	27 623 (73.6)	3843 (71.5)
> 4000 g	52 121 (13.8)	38 923 (12.2)	2045 (13.3)	9667 (25.7)	1486 (27.6)
Weight deviations, *n* (%) Missing, *n* = 2478					
SGA^b^	35 841 (9.5)	31 114 (9.8)	1416 (9.2)	3021 (8.0)	290 (5.4)
LGA^c^	9169 (2.4)	5892 (1.9)	937 (6.1)	2073 (5.5)	267 (5.0)
Vacuum or forceps, *n* (%)	56 555 (15.0)	56 033 (17.6)	0	0	522 (9.7)
Inducted, *n* (%)	53 185 (14.1)	40 471 (12.7)	1925 (12.5)	9752 (26.0)	1037 (19.3)
Stillborn, *n* (%)	719 (0.2)	629 (0.2)	33 (0.2)	48 (0.1)	9 (0.2)
Interpregnancy interval (year), *n* (%)					
≤ 1 year	1629 (0.4)	1432 (0.5)	55 (0.4)	125 (0.3)	17 (0.3)
> 1 year	374 785 (99.6)	316 685 (99.5)	15 318 (99.6)	37 422 (99.7)	5360 (99.7)
Prior cervical Conisation, *n* (%)	3623 (1.0)	3021 (0.9)	183 (1.2)	354 (0.9)	65 (1.2)

Abbreviations: BMI, body mass index; DM1, type 1 diabetes; DM2, type 2 diabetes; GDM, gestational diabetes mellitus; IUGR, intrauterine growth restriction; LGA, large for gestational age; SGA, small for gestational age.

^a^ Available from year 2004.

^b^ Small‐for‐gestational age (SGA) ≤ −1.28 SD under mean according to gender and gestational age.

^c^ Large‐for‐gestational age (LGA) > 1.28 SD over mean according to gender and gestational age.

Table 2 presents the frequency of sPTB in the subsequent pregnancy according to birth mode at the index birth. In women with one previous birth at term, the overall rate of sPTB in the subsequent pregnancy was 1.7%. Rates of subsequent preterm birth according to mode of index birth were as follows: 1.7% after vaginal index birth, 1.5% after prelabour CB, 1.7% after first stage CB and 2.3% after second‐stage CB, respectively. Second‐stage CB was associated with an increased risk of sPTB compared to vaginal birth (aOR 1.46, 95% CI 1.21–1.77). This association also included sPTB before GA 34 weeks and 32 weeks with aORs of, respectively, 1.75 (95% CI 1.22–2.54) and 1.66 (95% CI 1.01–2.72). Considering sPTB between GA 32 + 0–36 + 6 second‐stage CB was associated with a 1.76‐fold increased risk of sPTB (aOR 1.76, 95% CI 1.22–2.54). First stage CB was not associated with an increased risk of sPTB compared to vaginal birth.

**TABLE 2:** Risk of spontaneous preterm birth in a subsequent pregnancy by mode of index birth.

	Index birth												
		All *n* = 376 414	Vaginal *n* = 318 117		Prelabour CB *n* = 15 373			First stage CB *n* = 37 547			Second‐stage CB *n* = 5377		
		*n* (%)	*n* (%)		*n* (%)	OR (95% CI)	aOR (95% CI)	*n* (%)	OR (95% CI)	aOR (95% CI)	*n* (%)	OR (95% CI)	aOR (95% CI)
	Preterm birth												
Subsequent birth	< 37	6386 (1.7)	5399 (1.7)	Ref	230 (1.5)	0.88 (0.77–1.00)	0.87 (0.75–1.01)	634 (1.7)	0.99 (0.92–1.08)	1.03 (0.94–1.12)	123 (2.3)	1.35 (1.13–1.61)	1.46 (1.21–1.77)
	< 34	1533 (0.4)	1285 (0.4)	Ref	57 (0.4)	0.92 (0.70–1.20)	0.95 (0.71–1.27)	156 (0.4)	1.03 (0.87–1.21)	1.03 (0.85–1.25)	35 (0.7)	1.61 (1.15–2.25)	1.75 (1.22–2.54)
	< 32	902 (0.2)	755 (0.2)	Ref	29 (0.2)	0.79 (0.55–1.15)	0.82 (0.54–1.25)	97 (0.3)	1.09 (0.88–1.34)	1.09 (0.85–1.39)	21 (0.4)	1.65 (1.07–2.54)	1.66 (1.01–2.72)
	< 28	444 (0.1)	361 (0.1)	Ref	17 (0.1)	0.97 (0.60–1.58)	0.90 (0.51–1.56)	47 (0.1)	1.10 (0.81–1.49)	1.12 (0.80–1.57)	11 (0.2)	1.80 (0.99–3.28)	1.56 (0.77–3.14)
	Late preterm												
	32 + 0–36 + 6	5642 (1.5)	4644 (1.5)	Ref	201 (1.3)	0.90 (0.78–1.03)	0.95 (0.70–1.27)	537 (1.4)	0.98 (0.90–1.07)	1.03 (0.85–1.25)	102 (1.9)	1.30 (1.07–1.58)	1.76 (1.22–2.54)
	Very preterm												
	28 + 0–31 + 6	480 (0.1)	394 (0.1)	Ref	12 (0.1)	0.63 (0.36–1.12)	0.75 (0.40–1.40)	50 (0.1)	1.08 (0.80–1.44)	1.05 (0.73–1.51)	10 (0.2)	1.50 (0.80–2.81)	1.77 (0.88–3.57)
	Extreme preterm												
	22 + 0–27 + 6	444 (0.1)	361 (0.1)	Ref	17 (0.1)	0.97 (0.60–1.58)	0.90 (0.51–1.56)	47 (0.1)	1.10 (0.81–1.49)	1.12 (0.80–1.57)	11 (0.2)	1.80 (0.99–3.28)	1.56 (0.77–3.14)

*Note:* aOR: OR adjusted for maternal age > 40 years, BMI > 30 kg/m^2^, smoking status, hypertensive disorders, diabetic disorders in the subsequent pregnancy and interpregnancy interval < 12 months.

Abbreviations: aOR, adjusted odds ratio; CB, caesarean birth; CI, confidence interval; OR, odds ratio.

**FIGURE 2:**
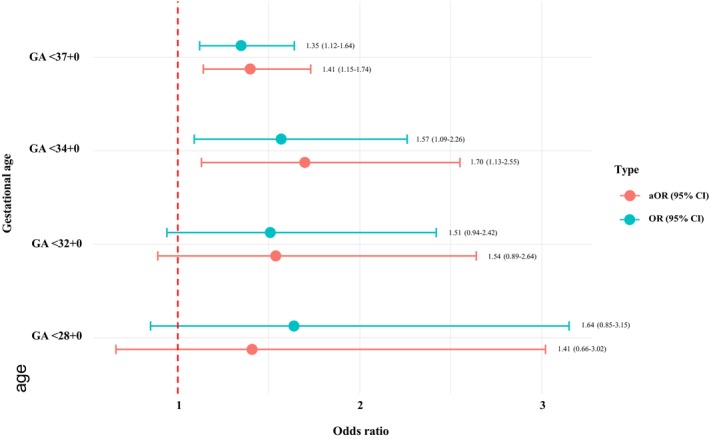
Risk of spontaneous preterm birth in a subsequent pregnancy by comparing second stage to first stage caesarean birth.

Compared to first stage CB, second‐stage CB was associated with an increased risk of subsequent sPTB (aOR 1.41, 95% CI 1.15–1.74) and sPTB before 34 weeks (aOR 1.70, 95% CI 1.13–2.55) (Figure 2).

We did not find an association between birth method in the index pregnancy and the risk of other pregnancy complications in the subsequent pregnancy, including cervical cerclage, placenta praevia and preterm prelabour rupture of the membranes (PPROM) (Table S1).

## 5 Discussion

### 5.1 Main Findings

In this large Danish population‐based study, we found that second‐stage CB in the index birth was associated with a higher risk of sPTB in the subsequent pregnancy compared to vaginal birth as well as to first stage CB.

### 5.2 Strengths and Limitations

The present study has both strengths and limitations that should be taken into consideration. To our knowledge, it is the largest cohort to date to investigate the association between second‐stage CB and sPTB. It is based on high‐quality validated quality data including a national population of 376 414 women with data from 1997–2021 [24, 25]. A key strength is our ability to minimise bias by adjusting for well‐known confounders for sPTB, where the lack of adjustment has previously been criticised [28]. Additionally, a notable strength is that we were able to minimise confounding through exclusion criteria, based on data from the Danish registers, including factors known to be independently associated with the risk of sPTB, such as cerclage.

Our study may have limitations related to the selection of the population. We excluded women based on parameters from their index birth, resulting in a selected population, excluding over half a million births, which may lower the generalisability. Further, the included registries only provide data on births after gestational week 22 + 0, which results in a lack of information on mid‐trimester pregnancy loss in our cohort; the risk of sPTB is low, and with stratification on gestational age and birth mode in the index delivery, the number of sPTB before 32 weeks is limited. This should be considered when interpreting the results. A major limitation was the lack of data on cervical dilatation at the time of the CB, as well as details on the time from full dilatation to birth and foetal head descent. These data are not available in DMBR, which is why we identified women with full dilatation CB using a proxy based on failed attempts of operative vaginal birth and prolonged second stage of labour. Consequently, we may have missed cases with CB in the second stage, which could potentially have led to an underestimation of the risk of sPTB after second‐stage CB.

Further, register‐based data from DMBR rely on specific procedural codes recorded by clinical staff, which introduce a risk of misclassification bias.

### 5.3 Interpretation

The overall rate of sPTB in a subsequent pregnancy was very low (1.7%) in our selected population of women with a previous term birth. Einum et al. reported a sPTB rate of 2.8% in a similar Norwegian population [23]. In contrast to this, Wood et al. reported in a study from Canada a sPTB rate of 2.7%–5.3% where Woolner et al. reported an overall sPTB rate of 3.6% in a subsequent pregnancy in a Scottish population [7, 8]. The variation is most likely explained by the generally low overall risk of PTB in the Nordic countries. Additional variations may be attributed to differences in maternal care, preventive interventions, birth management, CB guidelines or other cultural differences.

Compared to a vaginal index birth, we found a 1.46‐fold increased risk of sPTB in a subsequent pregnancy following second‐stage CB. This is in accordance with prior studies where increased risks of 1‐ to 5‐fold have been reported [7, 8, 11, 12, 13, 14]. These studies vary in inclusion criteria and sample size, ranging from 887 to 189 021 births [8, 12]. Wood et al. reported a RR of 1.6 in a Canadian cohort of 189 021 births, while Woolner et al. observed an increased risk of 5‐fold in a Scottish population of 30 253 births [7, 8]. The Canadian data closely aligned to our findings, whereas differences in the Scottish study are most likely due to variations in the background population, study period, sample size and possible differences in antenatal care over time. Notably, smaller sample sizes tend to report higher risks of sPTB after second‐stage CB, with risk estimates spanning from 2.40–5.37‐fold [7, 11, 12, 14]. Conversely, larger cohort studies (including 189 021 to 298 901 births) generally report risks ranging from no association to a 1.6‐fold increase [8, 22, 23]. The largest studies from Norway and the Netherlands did not distinguish between first‐ and second‐stage CB, which compromises the comparability to the findings for risk estimates for second‐stage CB [22, 23].

When we compared second‐stage CB to first stage CB, we found that second‐stage CB was associated with an increased risk of 1.41‐fold for sPTB and 1.70‐fold for sPTB < 34 weeks. These findings align with existing evidence from most studies with a reported increase in risk from 1.6 to 2‐fold [7, 8, 15]. In contrast, one study by Levine et al. stands out with a markedly higher risk in a retrospective cohort study with a 5.8‐fold increase of sPTB [12]. Levine et al. had a relatively small sample size of 887 births from a single hospital in the USA over a five‐year period, which may have added to a much stronger association [12].

Research suggests that the duration of the second stage of labour, combined with second‐stage CB, contributes to an increased risk of sPTB [14, 16, 29]. Additionally, foetal head station at the time of CB has been linked to a higher sPTB risk [30]. The key knowledge gap lies in determining whether, and if so which, women with a second‐stage CB should be offered interventions to prevent sPTB. Future studies could examine second‐stage CB and sPTB risk in subgroups based on additional obstetric interventions (e.g., vacuum, forceps), surgical complications (e.g., uterine tears, low uterotomy) and detailed labour characteristics such as duration of labour, cervical dilation, foetal station and mode of birth. Also, justification of screening for cervical insufficiency in women with a previous second‐stage CB should be evaluated, and the effects of the CB surgical technique should be further investigated.

## 6 Conclusion

In this large, nationwide register‐based cohort study, we found that a term second‐stage CB in a first pregnancy was associated with an increased risk of sPTB in a subsequent pregnancy when compared to vaginal birth and first stage CB. Further investigations of the performance of sPTB risk screening in women with a history of second‐stage CB as well as the impact of obstetric interventions, surgical complications and detailed labour characteristics in the previous birth are needed.

## Author Contributions

This study was initiated by S.A., L.K. and M.J. S.A., M.J., L.K. and C.H.H. formulated the research question and analytic strategy. S.A. and M.J. contributed to data management and made the statistical analysis. S.A. wrote the manuscript draft. L.K., M.J., C.H.H., J.G. and L.K.H. contributed to the interpretation of the results, reviewed the manuscript and approved the final draft. All authors accept responsibility for the final manuscript.

## Conflicts of Interest

The authors declare no conflicts of interest.

## Supporting information


**Figure S1.** Hierarchical categorising of obstetric codes for mode of index birth

**Table S1.** Risk of complications in a subsequent pregnancy by mode of index birth

## Data Availability

The data that support the findings of this study are available on request from the corresponding author. The data are not publicly available due to privacy or ethical restrictions.
