# Supplementary material for: Risk of Spontaneous Preterm Birth in a Subsequent Pregnancy After Full Dilatation Caesarean Birth: A Nationwide Cohort Study
Source: BJOG. 2025 May 28;132(11):1585–93. doi: 10.1111/1471-0528.18225 (PMC12411653; doi:10.1111/1471-0528.18225)
Supplement: Supplementary file 1 — Figure S1. Hierarchical categorising of obstetric codes for mode of index birth [file BJO-132-1585-s001.docx]

**Figure S1: Hierarchical categorising of obstetric codes for mode of index birth**

KMCA11, KMCA10A or KMCA10B

and no KMCA10E or vacuum or forceps

KMCA12, KMCA10D, KMCA10E and no KMAE20, KMAF20, DO665 or DO631 and no second stage CB and no vacuum or forceps

KMCA12, KMCA10D, KMCA10E and KMAE20 or KMAF20 or DO665 or DO631 or vacuum or forceps

**Prelabour CB**

**First stage CB**

**Second stage CB**

| **Procedural codes** | **Description** |
| --- | --- |
| KMCA11 | Prelabour caesarean birth |
| KMCA12 | Caesarean birth in labour |
| KMCA10A | Acute caesarean birth prelabour |
| KMCA10B | Caesarean birth planned prelabour |
| KMCA10D | Caesarean birth in labour |
| KMCA10E | Acute caesarean birth in labour |
| KMAE20 | Unsuccessful attempt of vacuum extraction |
| KMAF20 | Unsuccessful attempt of forceps |
| DO665 | Unsuccessful attempt of birth by use of vacuum extraction or forceps |
| DO631 | Caesarean birth after prolonged second stage of labour |

| **Definition** | **Description** |
| --- | --- |
| Vacuum extraction | KMAE00 or KMAE03 or DO81 |

CB, caesarean birth
