# Supplementary material for: Risk of Spontaneous Preterm Birth in a Subsequent Pregnancy After Full Dilatation Caesarean Birth: A Nationwide Cohort Study
Source: BJOG. 2025 May 28;132(11):1585–93. doi: 10.1111/1471-0528.18225 (PMC12411653; doi:10.1111/1471-0528.18225)
Supplement: Supplementary file 2 — Table S1. Risk of complications in a subsequent pregnancy by mode of index birth [file BJO-132-1585-s002.docx]

x

**Table S1: Risk of complications in a subsequent pregnancy by mode of index birth**

CB, caesarean birth; OR, odds ratio; aOR, adjusted odds ratio; CI, confidence interval.

aOR: Adjusted for maternal age <40 years, BMI >30, smoking status, hypertensive disorders, diabetic disorders and interpregnancy interval <12 months in the subsequent pregnancy.

| **Subsequent birth** | **Index birth** | | | | | | | | | | | | |
| --- | --- | --- | --- | --- | --- | --- | --- | --- | --- | --- | --- | --- | --- |
|  |  | **All** | **Vaginal** | | **Prelabour CB** | | | **First stage CB** | | | **Second stage CB** | | |
|  | **All, n (%)** | 376,414 (100) | 318,117 (84.5) | | 15,373 (4.1) | | | 37,547 (10.0) | | | 5,377 (1.4) | | |
|  |  | **n (%)** | **n (%)** |  | **n (%)** | **OR (95% CI)** | **aOR (95% CI)** | **n (%)** | **OR (95% CI)** | **aOR (95% CI)** | **n (%)** | **OR (95% CI)** | **aOR (95% CI)** |
|  | **Cerclage** | | | | | | | | | | | | |
|  |  | 3,180 (0.8) | 2,819 (0.9) | Ref | 131 (0.9) | 0.96 (0.81-1.14) | 1.01 (0.84-1.22) | 208 (0.6) | 0.62 (0.54-0.72) | 0.66 (0.57-0.76) | 22 (0.4) | 0.46 (0.30-0.70) | 0.53 (0.35-0.81) |
|  | **Placenta previa** | | | | | | | | | | | | |
|  |  | 1,124 (0.3) | 873 (0.3) | Ref | 82 (0.5) | 1.95 (1.55-2.44) | 1.86 (1.47-2.35) | 151 (0.4) | 1.47 (1.23-1.74) | 1.41 (1.17-1.68) | 18 (0.3) | 1.22 (0.76-1.95) | 1.11 (0.67-1.82) |
|  | **PPROM** | | | | | | | | | | | | |
|  |  | 3,931 (1.0) | 3,285 (1.0) | Ref | 171 (1.1) | 1.08 (0.92-1.25) | 1.05 (0.89-1.25) | 424 (1.1) | 1.09 (0.99-1.21) | 1.05 (0.94-1.18) | 51 (1.0) | 0.92 (0.70-1.21) | 0.87 (0.64-1.18) |
|  | **Stillborn** | | | | | | | | | | | | |
|  |  | 554 (0.2) | 452 (0.1) | Ref | 37 (0.2) | 1.70 (1.21-2.37) | 1.51 (1.03-2.23) | 60 (0.2) | 1.12 (0.86-1.47) | 1.14 (0.85-1.53) | 5 (0.1) | 0.65 (0.27-1.58) | 0.64 (0.24-1.70) |
